# Multimodal endoscopic diagnosis in biliary oncology: integrating EUS-guided tissue acquisition and cholangioscopy for personalized cancer care

**DOI:** 10.3389/fgstr.2026.1924535

**Published:** 2026-09-23

**Authors:** Petko Karagyozov, Nadica Male, Kalina Panova, Stela Nikolova

**Affiliations:** 1 Clinic of Gastroenterology, Acibadem City Clinic University Hospital Tokuda, Sofia, Bulgaria; 2 Medical University - Pleven, Pleven, Bulgaria

**Keywords:** cholangiocarcinoma, cholangioscopic biopsy, endoscopic ultrasound, personalized cancer therapy, precision oncology

## Abstract

The diagnosis of biliary malignancies remains challenging because of the heterogeneous growth patterns of cholangiocarcinoma, the limited sensitivity of conventional tissue acquisition techniques, and the growing need for molecular profiling in the era of precision oncology. Over the past decade, endoscopic ultrasound (EUS) and digital single-operator cholangioscopy have transformed the diagnostic approach by providing complementary information. EUS enables high-resolution imaging, locoregional staging, and tissue acquisition from primary tumors and regional lymph nodes, while cholangioscopy offers direct intraductal visualization and targeted biopsy of suspicious biliary lesions. This review summarizes the current evidence on the diagnostic performance, strengths, and limitations of EUS-guided tissue acquisition and cholangioscopy, emphasizing their complementary role within contemporary diagnostic algorithms. We also discuss emerging technologies, including molecular profiling, next-generation sequencing, artificial intelligence, radiomics, digital pathology, and liquid biopsy, which are shaping the future of biliary oncology. In addition to the available evidence, we present our institutional experience comparing EUS-guided tissue acquisition and cholangioscopy-directed biopsy in patients with suspected malignant biliary strictures. Our institutional experience supports the complementary use of EUS-guided tissue acquisition and cholangioscopy within a multimodal diagnostic approach. In the subgroup of patients who underwent both procedures, we observed high diagnostic sensitivity while maintaining an acceptable safety profile, supporting the potential value of a multimodal diagnostic approach. Integrating advanced endoscopic techniques with precision diagnostics is likely to optimize diagnostic accuracy and guide personalized management of biliary malignancies.

## 1 Introduction

Cholangiocarcinoma (CCA) is the second most common primary liver cancer after hepatocellular carcinoma and accounts for about 3% of all gastrointestinal cancers (1). Although relatively rare, its worldwide incidence has increased in recent decades, especially for intrahepatic cholangiocarcinoma, while perihilar tumors remain the most common anatomical subtype (2). Because of its aggressive nature, subtle clinical signs, and tendency to be diagnosed at an advanced stage, CCA remains associated with poor long-term survival (2). In routine clinical practice, most patients present with symptomatic biliary obstruction secondary to locally advanced disease, leaving only a minority eligible for potentially curative surgical resection (1, 3). Although surgical resection and, in highly selected cases, liver transplantation offer the best chance for long-term survival, most patients require systemic therapy, which remains primarily palliative due to the limited chemosensitivity of cholangiocarcinoma (3, 4).

The diagnosis of biliary malignancies remains one of the greatest challenges in gastrointestinal oncology (5). The macroscopic growth pattern of cholangiocarcinoma varies considerably according to its anatomical location and has a direct impact on diagnostic evaluation. Intrahepatic tumors may present as mass-forming, periductal infiltrating, or intraductal growing lesions, whereas perihilar and distal cholangiocarcinomas typically present as infiltrative sclerosing strictures with longitudinal subepithelial extension and, less commonly, as intraductal papillary tumors. This morphological diversity contributes to the diagnostic complexity of biliary malignancies and indicates the need for complementary endoscopic techniques that can assess both extramural disease and intraductal pathology (6). These features often result in subtle or nonspecific findings on cross-sectional imaging while simultaneously limiting the diagnostic yield of conventional tissue acquisition techniques (6). Despite substantial advances in cross-sectional imaging and endoscopic technologies, accurately differentiating benign from malignant biliary strictures is still a major clinical challenge (5). Furthermore, the growing implementation of an individualized approach to cancer care has increased demand for high-quality tissue specimens, as molecular characterization of cholangiocarcinoma is now essential for prognostic assessment and the selection of targeted treatment (3, 7).

Conventional ERCP-guided sampling techniques have long represented the foundation of pathological diagnosis in patients with suspected biliary malignancy. However, their limited sensitivity- approximately 45% for brush cytology and 48% for intraductal biopsies - means that many malignant strictures remain undiagnosed despite repeated tissue acquisition. Although combining both modalities modestly increases diagnostic yield, overall sensitivity remains below 60%, pointing to the need of advanced endoscopic techniques that yield higher-quality tissue and increase diagnostic confidence (8). Consequently, negative cytological or histological findings cannot accurately exclude malignancy, frequently necessitating repeated procedures or multimodal diagnostic evaluation before definitive therapeutic decisions can be made.

Over the last decade, remarkable advances in interventional endoscopy have fundamentally changed the diagnostic approach to biliary oncology. Endoscopic ultrasound (EUS) has evolved from a purely imaging modality into a comprehensive approach for high-resolution loco-regional assessment, tissue acquisition, advanced nodal staging, and increasingly, molecular characterization of biliary tumors (9). The broad use of fine-needle biopsy (FNB) has enabled the acquisition of core tissue specimens suitable not only for histopathological diagnosis but also for immunohistochemistry and next-generation sequencing, facilitating the implementation of precision oncology (9–11). In parallel, digital single-operator cholangioscopy has changed intraductal evaluation by allowing direct visualization of biliary mucosal abnormalities and targeted biopsy of suspicious lesions, thereby overcoming many limitations of conventional ERCP-guided sampling (12, 13).

Importantly, EUS and cholangioscopy should not be viewed as competing diagnostic modalities but rather as complementary techniques that assess different biological and anatomical aspects of biliary malignancies (13). While EUS is excellent in identifying extramural disease, regional lymph node involvement, vascular invasion, and lesions beyond the bile duct wall, cholangioscopy uniquely provides direct intraductal visualization and targeted sampling of mucosal pathology (9, 12). The combined use of EUS and cholangioscopy has changed the evaluation of biliary malignancies by providing complementary diagnostic information that improves tissue diagnosis and helps guide treatment decisions (13).

In this review, we discuss the complementary roles of EUS-guided tissue acquisition and cholangioscopy in the diagnosis of biliary malignancies, review the current evidence, and present our single-center experience comparing EUS-guided sampling with cholangioscopy-directed biopsies in patients with suspected malignant biliary strictures. Through this integrated perspective, we aim to demonstrate how multimodal endoscopic evaluation can improve diagnostic certainty and support clinical decision-making in patients with biliary malignancies.

## 2 Diagnostic challenges in biliary malignancies

### 2.1 Biology and morphological subtypes of cholangiocarcinoma

Cholangiocarcinoma (CCA) is a heterogeneous malignancy with distinct biological and morphological characteristics that directly influence its diagnosis, staging, and management. Based on its anatomical location, CCA is classified into intrahepatic (iCCA), perihilar (pCCA), and distal (dCCA), each representing a distinct clinical entity with distinct patterns of growth, molecular alterations, and therapeutic implications (**Figure 1**) (14).

**Figure 1:**
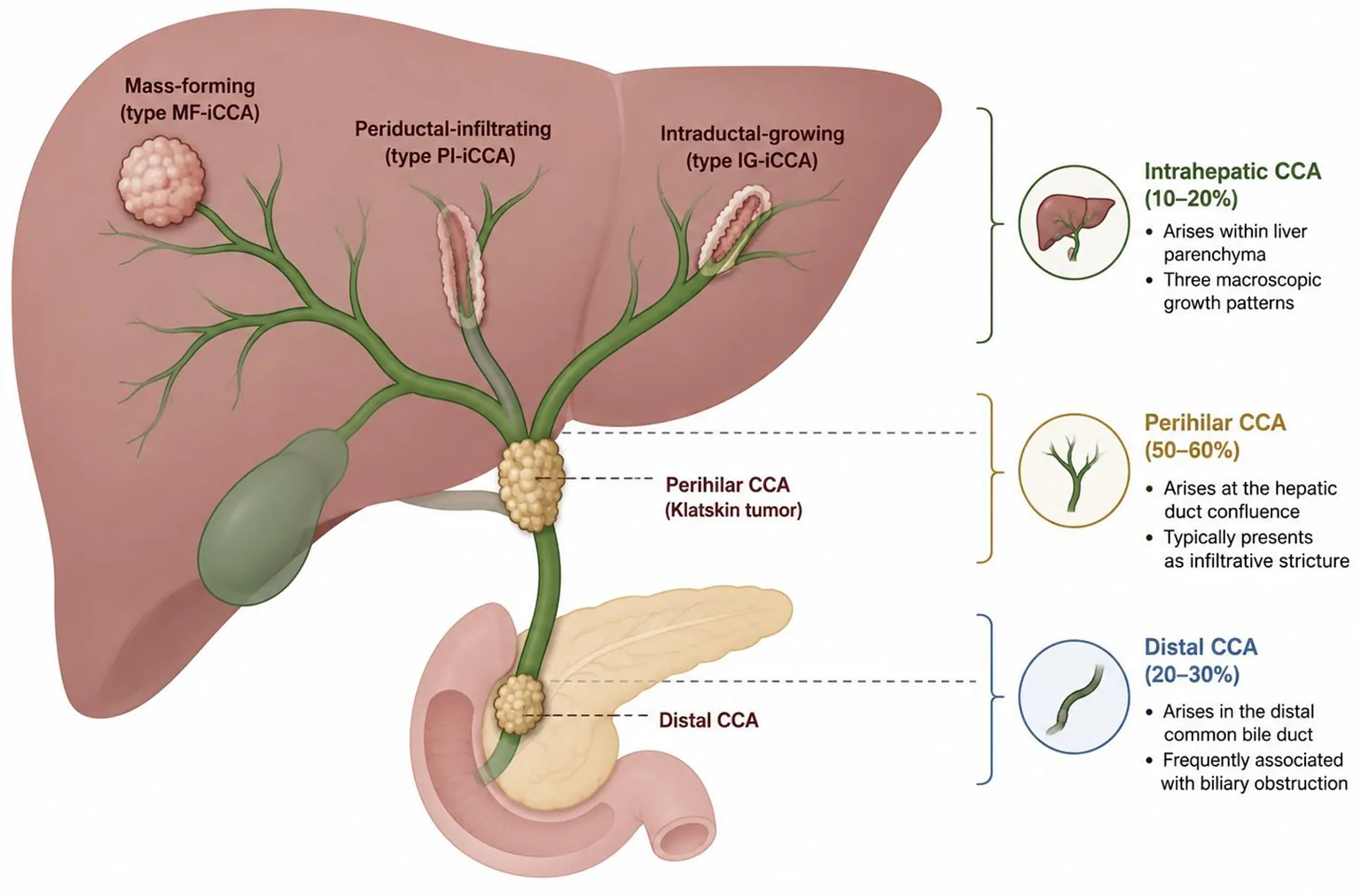
Anatomical classification and macroscopic growth patterns of cholangiocarcinoma. (The reported percentages represent the approximate relative frequency of the different cholangiocarcinoma subtypes according to published epidemiological data).

Intrahepatic cholangiocarcinoma demonstrates three principal macroscopic growth patterns: mass-forming, periductal infiltrating, and intraductal growing (15, 16). The mass-forming subtype is the most common and typically presents as a well-defined hepatic lesion arising from small intrahepatic bile ducts. In contrast, periductal infiltrating tumors extend longitudinally along the bile duct wall, producing irregular biliary strictures without necessarily forming a clear mass. The intraductal growing subtype is characterized by papillary proliferation within the bile duct lumen and is less frequently encountered (15, 16).

Perihilar and distal cholangiocarcinomas differ from intrahepatic tumors in that they most commonly exhibit an infiltrative sclerosing growth pattern with longitudinal subepithelial extension along the bile duct. As a result, these tumors frequently present as indeterminate biliary strictures rather than mass lesions, making histological confirmation particularly challenging (17). Less commonly, they may develop as intraductal papillary tumors with more conspicuous endoscopic findings (16).

The marked heterogeneity of cholangiocarcinoma significantly affects endoscopic diagnosis. Mass-forming tumors are generally well suited for EUS-guided tissue acquisition because they can be readily identified and sampled under ultrasound guidance (18). Conversely, infiltrative lesions usually lack a clear mass and spread predominantly within the bile duct wall, reducing the diagnostic yield of EUS-guided biopsy while favoring direct intraductal evaluation with cholangioscopy (13). Similarly, papillary intraductal tumors are more effectively assessed by cholangioscopy, which allows direct visualization of the biliary mucosa and targeted biopsy of suspicious lesions (13).

As summarized in **Table 1**, the anatomical location and growth pattern of cholangiocarcinoma largely determine the optimal endoscopic diagnostic strategy (13, 20). These differences emphasize that the diagnostic performance of EUS-guided tissue acquisition and cholangioscopy is largely determined by tumor morphology and anatomical location. Rather than competing techniques, they provide complementary information by evaluating different components of the disease (22). Knowing these biological and anatomical characteristics is therefore essential for selecting the most appropriate endoscopic approach as well as optimizing tissue acquisition in patients with suspected biliary malignancy.

**Table 1:** Relationship between cholangiocarcinoma morphology and the preferred endoscopic diagnostic approach.

Tumor subtype	Predominant morphology	Diagnostic challenge	Preferred endoscopic approach
Mass-forming iCCA	Solid hepatic mass	Easy EUS target	**EUS-guided FNB**
Periductal infiltrating iCCA	Longitudinal ductal spread	No discrete mass	**EUS + cholangioscopy**
Intraductal growing iCCA	Papillary intraductal lesion	Superficial mucosal disease	**Cholangioscopy**
Perihilar CCA	Infiltrative hilar stricture	Subepithelial extension	**Combined EUS and cholangioscopy**
Distal CCA	Distal biliary stricture ± mass	Variable morphology	**EUS-guided tissue acquisition ± cholangioscopy**

(Adapted and synthesized from Banales et al., WHO Classification of Digestive System Tumors, ESGE and ASGE guideline) (13, 19–21).

Bold values indicate the preferred diagnostic approach for each morphological subtype.

### 2.2 Why tissue diagnosis remains difficult?

Histological confirmation remains the foundation of cholangiocarcinoma diagnosis, directing treatment decisions and, increasingly, molecular profiling for individualized therapies (3, 7). However, obtaining adequate tissue from biliary lesions is often technically challenging because of the unique biological and anatomical characteristics of these tumors. Unlike many gastrointestinal malignancies, which form discrete masses, cholangiocarcinoma is characterized by abundant desmoplastic stroma that often constitutes a substantial proportion of the tumor volume (23). This dense stromal reaction is believed to arise from chronic biliary injury and repair mechanisms and is composed of cancer-associated fibroblasts, endothelial cells, inflammatory immune cells, and an extensive extracellular matrix rich in collagen, laminin, and fibronectin (24). Beyond supporting tumor growth and progression, the desmoplastic microenvironment has important diagnostic implications. The marked fibrosis and relatively low density of malignant cells often yield inadequate or nonrepresentative tissue samples, contributing to the limited sensitivity of conventional sampling techniques (23). Consequently, obtaining high-quality tissue often requires advanced endoscopic approaches that target both the tumor and its surrounding microenvironment (25).

The evaluation of indeterminate biliary strictures begins with noninvasive imaging, which plays a pivotal role in detecting biliary obstruction, localizing the stricture, and assessing the extent of the disease. Abdominal ultrasonography is widely available and is often the first-line imaging modality, readily demonstrating biliary dilatation proximal to the obstruction. However, its ability to identify the underlying etiology of the stricture is limited (25). Cross-sectional imaging with computed tomography (CT) and magnetic resonance imaging with magnetic resonance cholangiopancreatography (MRI/MRCP) provides a more comprehensive anatomical assessment. MRI/MRCP is generally considered superior to CT for characterizing biliary strictures and delineating the biliary anatomy. The reported sensitivity and specificity for Magnetic Resonance Cholangiopancreatography (MRCP) versus multi-detector CT are 88% and 95% versus 75% and 60%, respectively (26).

ERCP remains the cornerstone of endoscopic tissue acquisition in patients with suspected cholangiocarcinoma. During cholangiography, malignant strictures typically appear as long, irregular, and asymmetric narrowing of the bile duct, although these features are not sufficiently specific to establish a definitive diagnosis. Tissue acquisition is traditionally performed using brush cytology and fluoroscopy-guided intraductal forceps biopsy (27). Brush cytology is technically simple, safe, and widely available, whereas forceps biopsy provides larger tissue samples but may be technically challenging, particularly in proximal or complex hilar strictures.

Despite their widespread use, both techniques have limited diagnostic sensitivity. In a landmark meta-analysis by Navaneethan et al. (28), including nine studies, the pooled sensitivity was 45% (95% CI, 40–50%) for brush cytology and 48% (95% CI, 42.8–53.4%) for intraductal biopsy, while both methods maintained excellent specificity of approximately 99%. Combining brush cytology with forceps biopsy resulted in only a modest increase in sensitivity to 59%, highlighting the inherent limitations of conventional ERCP-based sampling in patients with malignant biliary strictures (8).

Several adjunctive techniques have been developed to improve the diagnostic performance of ERCP-guided sampling. Fluorescence *in situ* hybridization (FISH), which detects chromosomal abnormalities associated with biliary malignancies, has been shown to significantly increase the sensitivity of routine cytology. Barr Fritcher et al. (28) demonstrated that adding FISH increased the sensitivity of brush cytology from approximately 20% to 43%, while the combination of brush cytology, intraductal biopsy, and FISH achieved diagnostic sensitivities approaching 82% in selected patient populations (28).

The introduction of digital single-operator cholangioscopy systems, such as Spyglass™ DS, has enabled direct endoscopic visualization of the biliary tree and targeted biopsy of suspicious lesions. By allowing tissue acquisition under direct visual guidance, cholangioscopy overcomes several limitations of fluoroscopy-guided sampling and improves diagnostic confidence in patients with indeterminate biliary strictures (27). The reported sensitivity of cholangioscopy-directed biopsies is 64% (27), supporting its role as a valuable adjunct when conventional ERCP-guided brushings and forceps biopsies are nondiagnostic (29).

Several factors contribute to the limited diagnostic performance of conventional ERCP-based sampling. Brush cytology often retrieves only superficial epithelial cells, while forceps biopsies are constrained by their small size and the technical difficulty of accurately targeting infiltrative lesions under fluoroscopic guidance (30). Furthermore, the pronounced fibrotic reaction characteristic of cholangiocarcinoma, particularly in perihilar tumors, reduces cellular yield and increases the likelihood of nondiagnostic or false-negative specimens (23). These limitations are even more pronounced in patients with primary sclerosing cholangitis or chronic inflammatory biliary disease, where reactive epithelial changes may closely resemble malignancy (30).

The inherent limitations of conventional tissue acquisition demonstrate the need for complementary diagnostic techniques. EUS-guided tissue acquisition provides access to extramural lesions, regional lymph nodes, and mass-forming tumors (9) while digital cholangioscopy allows direct visualization of the biliary mucosa and targeted biopsy of suspicious intraductal lesions (12). Because these modalities evaluate different anatomical components of biliary malignancies, neither should be considered a replacement for the other. Instead, their complementary use offers the greatest potential to increase diagnostic accuracy, reduce false-negative results, and facilitate timely therapeutic decision-making (18).

## 3 EUS-guided tissue acquisition in biliary oncology

### 3.1 Evolution of EUS in biliary cancer

Over the past three decades, endoscopic ultrasound (EUS) has evolved from a purely diagnostic imaging modality into an indispensable tool in the management of pancreatobiliary diseases. Initially introduced for high-definition visualization of the gastrointestinal wall and adjacent structures, echoendoscopy has benefited from continuous advances in echoendoscope technology, needle design, and interventional techniques, considerably expanding its role. Today, EUS is used not only for lesion detection and characterization but also for real-time tissue acquisition, locoregional staging, and minimally invasive therapeutic interventions (31).

In biliary oncology, EUS has become an integral part of the diagnostic pathway, particularly for patients with indeterminate biliary strictures or suspected cholangiocarcinoma. Research shows that EUS outperforms CT and MRCP in distinguishing malignant from benign biliary strictures, with reported sensitivities of 25% to 91% and specificities of 89% to 100% (31). Unlike conventional imaging modalities, EUS provides detailed anatomical assessment and enables tissue sampling from primary tumors, regional lymph nodes, and metastatic lesions in a single procedure (32). Beyond tissue acquisition, EUS provides high-resolution evaluation of tumor size, depth of invasion, vascular involvement, and regional lymph node status, all of which are important for accurate staging and assessment of surgical resectability. In addition, EUS-guided sampling of suspicious lymph nodes enables pathological confirmation of nodal metastases, further improving staging accuracy. Several studies have reported up to 85% accuracy in assessing vascular invasion, highlighting the important role of EUS in preoperative planning (33).

The role of EUS has expanded further with the introduction of fine-needle biopsy (FNB) needles, which provide core tissue specimens suitable for histopathological examination, immunohistochemistry, and molecular analysis. As targeted therapies become increasingly available for cholangiocarcinoma, EUS-guided tissue acquisition has progressed beyond establishing a histological diagnosis and now plays an essential role in precision oncology by providing adequate tissue for genomic profiling and biomarker testing (31).

Current international guidelines recommend EUS-guided tissue acquisition in selected patients with suspected biliary malignancy, particularly when a mass lesion is present, regional lymph node assessment is required, or prior ERCP-based sampling was nondiagnostic. Rather than replacing ERCP or cholangioscopy, EUS provides complementary diagnostic information by evaluating structures beyond the bile duct wall, making it a key element of the modern multimodal approach to biliary malignancies (13, 20, 33).

### 3.2 Imaging characteristics

Endoscopic ultrasound provides high-quality imaging of the biliary tree and surrounding structures, making it a fundamental tool for evaluating suspected biliary malignancies. Unlike cross-sectional imaging, EUS allows real-time assessment of the bile duct wall, adjacent vascular structures, regional lymph nodes, and surrounding organs (34). This close proximity to the biliary system allows detection of subtle pathological changes that may not be apparent on computed tomography (CT) or magnetic resonance imaging (MRI), particularly in small distal lesions and early-stage disease (31).

The sonographic appearance of cholangiocarcinoma varies according to its anatomical location and growth pattern. Mass-forming lesions typically appear as irregular hypoechoic masses with poorly defined margins, whereas infiltrative tumors are more commonly characterized by focal or diffuse bile duct wall thickening, luminal narrowing, and disruption of the normal ductal architecture. Periductal infiltrating tumors may be particularly difficult to identify because they frequently spread longitudinally along the bile duct without forming a definite mass, pointing out the importance of careful evaluation of ductal wall abnormalities (34).

Beyond lesion detection, EUS provides comprehensive assessment of locoregional tumor extension. High-definition imaging allows accurate evaluation of vascular invasion, involvement of adjacent organs, and regional lymph node enlargement, all of which are necessary for staging and determining surgical resectability (32).

Another important advantage of EUS is its ability to identify alternative causes of biliary obstruction. Pancreatic adenocarcinoma, ampullary neoplasms, metastatic lymphadenopathy, benign inflammatory strictures, and IgG4-related sclerosing cholangitis may all mimic cholangiocarcinoma on cross-sectional imaging (35).

### 3.3 EUS-guided tissue acquisition

The introduction of EUS-guided tissue acquisition has significantly expanded the diagnostic potential of endoscopic ultrasound, allowing simultaneous lesion characterization, locoregional staging, and histological confirmation (31). Unlike ERCP-based sampling, which is limited to the ductal lumen, EUS provides access to primary tumors, suspicious regional lymph nodes, and metastatic lesions under real-time ultrasound guidance. This comprehensive approach has established EUS as an essential component of the diagnostic work-up for suspected biliary malignancies (18).

The development of fine-needle biopsy (FNB) technology has further enhanced the role of EUS in biliary oncology. While fine-needle aspiration (FNA) primarily provides cytological material, FNB enables acquisition of core tissue with preserved architecture, facilitating histopathological examination, immunohistochemistry, and molecular analyses. Consequently, FNB is increasingly preferred when comprehensive tissue characterization or genomic profiling is anticipated, particularly in patients with unresectable or advanced disease (36).

In addition to facilitating tissue acquisition, EUS plays an important role in preoperative nodal staging. De Jong et al. reported that EUS greatly influenced clinical decision-making in patients with presumed resectable intrahepatic and mid-bile-duct cholangiocarcinoma by increasing detection of regional lymph node metastases. Although the impact was less evident in perihilar and distal tumors, the authors suggested that systematic EUS staging may further optimize patient selection for surgery (37).

The diagnostic yield of EUS-guided tissue acquisition varies according to tumor location. Distal cholangiocarcinomas generally present as discrete masses adjacent to the duodenum and are therefore readily accessible for biopsy. In contrast, perihilar tumors frequently exhibit an infiltrative growth pattern without a well-defined mass, thereby reducing tissue acquisition rates. In a prospective observational study by Mohamadnejad et al., EUS identified all distal cholangiocarcinomas and 83% of proximal tumors, while the sensitivity of EUS-guided tissue acquisition was significantly higher for distal than proximal lesions (81% vs. 59%) (38).

EUS additionally contributes to the early diagnosis of biliary malignancies when cross-sectional imaging is inconclusive. Sai et al. evaluated 142 patients with unexplained biliary dilatation and elevated cholestatic liver enzymes, demonstrating that combining EUS with magnetic resonance cholangiography increased diagnostic sensitivity from 80% to 90% and specificity from 90% to 98% compared with magnetic resonance cholangiography alone, thereby highlighting the complementary role of EUS in the diagnostic pathway (39).

Even with its advantages, EUS-guided tissue acquisition requires careful patient selection. Particular concern has been highlighted regarding transperitoneal sampling of potentially resectable or transplant-eligible perihilar cholangiocarcinoma because of the theoretical risk of tumor seeding (40). In a retrospective cohort of patients undergoing neoadjuvant chemoradiotherapy followed by liver transplantation, Heimbach et al. observed a significantly higher incidence of peritoneal metastases among patients with positive transperitoneal biopsies than among those who had not undergone biopsy (41). Although evidence specifically evaluating EUS-guided sampling remains limited, these data have led many transplant centers to avoid transperitoneal biopsy in candidates for liver transplantation. Therefore, tissue acquisition should always be individualized and discussed within a multidisciplinary team.

Overall, EUS-guided tissue acquisition has become a cornerstone of modern biliary oncology, combining high-definition imaging with accurate histological diagnosis and locoregional staging. When combined with other endoscopic modalities, EUS provides thorough diagnostic information that directly influences therapeutic decision-making.

Over the past two decades, numerous studies have demonstrated the high diagnostic performance of EUS-guided tissue acquisition in patients with suspected biliary malignancies. While the reported diagnostic yield varies according to tumor location, lesion morphology, and needle type, EUS consistently outperforms conventional ERCP-based sampling, particularly when a mass lesion is identified (42).

Current ESGE and ASGE recommendations advocate EUS-guided tissue acquisition as an integral component of the diagnostic work-up for suspected biliary malignancies when tissue confirmation is required (13, 20). In clinical practice, EUS is especially useful after nondiagnostic ERCP-based sampling, in patients with associated mass lesions, and when regional lymph node assessment or molecular profiling is anticipated.

### 3.4 Molecular profiling and precision oncology

The role of EUS-guided tissue acquisition has progressed beyond establishing a histopathological diagnosis. With the growing implementation of precision oncology, adequate tissue sampling has become increasingly important for complete molecular characterization, prognostic stratification, and selection of targeted therapies (43).

Comprehensive genomic profiling has demonstrated that cholangiocarcinoma is a molecularly heterogeneous disease characterized by various actionable genetic alterations. Among the most clinically relevant biomarkers are FGFR2 fusions, IDH1 mutations, HER2 amplification, BRAF V600E mutations, NTRK fusions, and microsatellite instability (MSI-H)/mismatch repair deficiency (dMMR) (44). Identification of these alterations has led to the development of precision therapies that have markedly expanded treatment options for selected patient populations (45, 46).

Compared with conventional cytology obtained by ERCP brushings or EUS-FNA, EUS-guided fine-needle biopsy provides core tissue specimens with preserved architecture, improving specimen adequacy for immunohistochemistry, next-generation sequencing (NGS), and other ancillary molecular analyses (47). Several studies have demonstrated that FNB yields a higher proportion of samples suitable for complete genomic profiling while decreasing the need for repeat tissue acquisition (47).

Current international guidelines recommend molecular profiling in patients with unresectable or metastatic cholangiocarcinoma whenever adequate tissue is available (33, 48). In this setting, EUS-guided tissue acquisition has become an important platform for precision oncology.

## 4 Cholangioscopy: direct intraductal assessment

Although EUS has transformed the evaluation of biliary malignancies, its diagnostic performance may be limited in infiltrative tumors that do not form a solid, defined mass. Digital single-operator cholangioscopy complements EUS by providing direct visualization of the biliary mucosa and allowing targeted tissue acquisition under real-time endoscopic guidance. This unique intraductal perspective is especially useful in assessing indeterminate biliary strictures, where conventional ERCP-based sampling is often nondiagnostic (49).

Unlike fluoroscopic imaging, cholangioscopy allows detailed evaluation of mucosal morphology and lesion extent. Characteristic features suggestive of malignancy include irregular mucosal nodularity, papillary projections, ulceration, irregular surface architecture, and dilated tortuous vessels, with abnormal neovascularization being one of the most reliable visual indicators (50). Although visual assessment alone cannot replace histological confirmation, it provides important diagnostic information and facilitates targeted biopsy of the most suspicious areas (49).

The diagnostic value of cholangioscopic visual assessment has been consistently demonstrated across multiple studies. A systematic review by Kulpatcharapong et al. (51) reported that the sensitivity and specificity of visual impression for differentiating malignant from benign biliary strictures ranged from 67% to 100% and 49% to 100%, respectively, depending on the cholangioscopy platform used. Notably, in patients with prior nondiagnostic ERCP-based tissue sampling, visual assessment achieved sensitivities of 90-100% and overall diagnostic accuracy approaching 97%, highlighting its value as a second-line diagnostic tool (51). Furthermore, a meta-analysis by de Oliveira et al. (52), including 283 patients undergoing digital single-operator cholangioscopy, demonstrated excellent diagnostic performance of visual impression alone, with a pooled sensitivity of 94%, specificity of 95%, and overall accuracy of 94% (52).

A major strength of digital single-operator cholangioscopy is the ability to obtain biopsies under direct visual guidance. By targeting the most suspicious areas of a biliary stricture, cholangioscopy minimizes sampling error and improves the quality of the acquired tissue compared with conventional fluoroscopy-guided techniques. This advantage is reflected in the available evidence. In a multinational registry, Almadi et al. (53) reported tissue adequacy in 93% of cases, with a sensitivity of 75%, specificity of 100%, and an overall diagnostic accuracy of 87% for cholangioscopy-guided biopsy (53). Similarly, a systematic review demonstrated consistently high diagnostic performance in patients undergoing initial evaluation for biliary strictures, with sensitivities ranging from 65% to 100% and specificities ranging from 75% to 100% (51). While prior nondiagnostic ERCP-based sampling is associated with a lower diagnostic yield, cholangioscopy-guided biopsy remains clinically valuable in this challenging setting. Although reported sensitivities range from 38% to 88%, specificity consistently exceeds 94%, allowing cholangioscopy to provide reliable histological confirmation in patients with persistent diagnostic uncertainty (51).

Despite these advantages, cholangioscopy has several limitations. The examination is confined to the biliary lumen and therefore cannot assess extramural tumor extension, vascular invasion, or regional lymph node metastases. In addition, biopsy specimens are relatively small and may be inadequate for extensive molecular analyses. Procedure-related adverse events, particularly cholangitis, also occur more frequently than after EUS-guided tissue acquisition (54, 55).

## 5 Integrating EUS and cholangioscopy: a multimodal diagnostic strategy

No single endoscopic technique can address all the diagnostic challenges encountered in biliary malignancies. Although EUS and digital cholangioscopy have each substantially improved the evaluation of indeterminate biliary strictures, they provide fundamentally different but complementary information. EUS enables detailed assessment of the extrabiliary component of the disease, including tumor extent, vascular invasion, regional lymph node involvement, and tissue acquisition from mass lesions. In contrast, cholangioscopy offers direct visualization of the biliary mucosa, allowing precise characterization of intraductal abnormalities and targeted biopsy of suspicious lesions. Rather than competing modalities, they should be considered complementary components of a comprehensive diagnostic strategy (55, 56).

Selecting the most appropriate endoscopic approach should be guided by the clinical presentation and the lesion’s morphological characteristics. Patients with a discrete mass lesion or suspected nodal disease derive particular benefit from EUS-guided tissue acquisition, whereas infiltrative strictures without an identifiable mass are often better evaluated with digital cholangioscopy. In many cases, combining both techniques provides the highest diagnostic confidence by overcoming the limitations of each modality.

### 5.1 Institutional experience: a retrospective single-center analysis

To illustrate the clinical value of this multimodal approach, we analyzed our institutional experience comparing EUS-guided tissue acquisition and cholangioscopy-directed biopsy in patients with suspected malignant biliary strictures.

#### 5.1.1 Study design

To evaluate the complementary diagnostic performance of EUS-guided tissue acquisition and digital cholangioscopy, we performed a retrospective analysis of consecutive patients who underwent endoscopic evaluation for suspected malignant biliary strictures at our tertiary referral center between January 2020 and September 2025. Patients without a definitive diagnosis, incomplete clinical data, or less than 6 months of follow-up in benign cases were excluded from the diagnostic sensitivity analysis.

The choice of diagnostic modality reflected routine clinical practice and was determined by multidisciplinary evaluation based on lesion morphology, anatomical location, cross-sectional imaging findings, and the anticipated diagnostic yield of each technique. EUS-guided tissue acquisition was preferentially performed in patients with a discrete mass lesion, suspected extrabiliary tumor extension, or suspicious regional lymph nodes suitable for sampling. Digital cholangioscopy was primarily used for indeterminate biliary strictures without a clearly defined mass lesion, particularly following nondiagnostic ERCP-based tissue acquisition or when detailed intraductal evaluation was required. A combined approach was reserved for selected patients in whom diagnostic uncertainty persisted or when complementary assessment of both intraductal and extrabiliary disease components was considered clinically beneficial. Consequently, allocation to each modality was individualized rather than predefined and should not be interpreted as a standardized comparative diagnostic protocol.

The final diagnosis of malignancy was established by histopathological confirmation obtained from surgical specimens or endoscopic tissue acquisition. Benign biliary strictures were confirmed by multidisciplinary assessment and a minimum of 6 months of clinical and radiological follow-up and were excluded from the diagnostic sensitivity analysis.

Sensitivity analyses were restricted to patients with confirmed malignancy. Because patients were not prospectively assigned to one diagnostic strategy, the reported diagnostic performance represents the observed yield of each modality within the clinical scenarios in which it was applied rather than a direct comparison between techniques.

A positive result was defined as histological confirmation of malignancy on EUS-guided biopsy or cholangioscopy-directed biopsy, or the presence of characteristic malignant features on cholangioscopic visual assessment. For patients who underwent both EUS and cholangioscopy, combined sensitivity was defined as a positive diagnosis obtained by either modality.

All digital cholangioscopy procedures were performed using the SpyGlass™ DS Direct Visualization System (Boston Scientific, Marlborough, MA, USA). Detailed procedural characteristics of EUS-guided tissue acquisition, including FNB needle type and gauge, were not consistently available because of the retrospective nature of the study.

Statistical analysis: Data were analyzed using IBM SPSS Statistics version 19.0. Continuous variables are presented as median (interquartile range [IQR]) and categorical variables as frequencies and percentages. Diagnostic sensitivity is reported with 95% confidence intervals (CIs).

#### 5.1.2 Results

A total of 117 consecutive patients underwent endoscopic evaluation for suspected biliary malignancy during the study period. Following surgical histopathology or multidisciplinary consensus based on imaging and clinical follow-up, 96 patients (82%) were confirmed to have malignant disease. The median age was 66 years (IQR, 58- 73), and 53% were male. Most patients had preserved performance status (ECOG 0-1, 94%). Perihilar lesions predominated, accounting for 78% of malignant strictures, while 20% were located in the distal bile duct. Among perihilar tumors, the majority were classified as Bismuth- Corlette type III or IV, reflecting the complexity of the cohort referred to our tertiary center.

EUS-guided fine-needle biopsy was performed in 40 patients with confirmed malignancy, primarily in the presence of a discrete mass lesion or suspicious regional lymph nodes. The procedure achieved a sensitivity of 65% (26/40; 95% CI, 46.3-76.3). Although the diagnostic yield was lower than that reported in the literature for distal cholangiocarcinoma, this likely reflects the predominance of infiltrative hilar lesions in our cohort, many of which lacked a clearly defined sonographic target.

Cholangioscopy-directed biopsy was performed in 72 malignant cases, achieving a sensitivity of 72.2% (52/72; 95% CI, 60.5-82.9). In addition to targeted tissue acquisition, direct visual assessment provided important diagnostic information. Characteristic cholangioscopic features suggestive of malignancy were identified in 61 of 72 patients, corresponding to a sensitivity of 84.7% (95% CI, 74.3- 92.3). These findings highlight the additional value of direct intraductal visualization, particularly in infiltrative strictures where obtaining representative tissue may be challenging.

The highest diagnostic performance was observed in the subgroup of 26 patients who underwent both EUS-guided tissue acquisition and digital cholangioscopy. When evaluated individually, each modality demonstrated moderate sensitivity; however, combining the two approaches substantially improved diagnostic performance. Overall sensitivity increased to 96.2% (25/26; 95% CI, 81.1- 99.9), with only a single malignant lesion remaining undiagnosed after the combined endoscopic evaluation. These findings emphasize the complementary nature of the two techniques and demonstrate that integrating extramural assessment with direct intraductal evaluation significantly reduces the risk of false-negative results.

Both procedures demonstrated an acceptable safety profile. Mild pancreatitis occurred following EUS-guided tissue acquisition in 4.5% of patients. Cholangioscopy was associated with a higher incidence of adverse events, including cholangitis (13.5%), pancreatitis (3.4%), and bleeding (2.2%); however, all complications were classified as mild and resolved with conservative treatment. No severe procedure-related adverse events or procedure-related mortality were observed.

#### 5.1.3 Discussion of institutional findings

Our institutional experience supports the evolving concept that EUS-guided tissue acquisition and digital cholangioscopy should be viewed as complementary rather than competing diagnostic modalities for evaluating biliary malignancies. While each technique demonstrated moderate diagnostic sensitivity when used individually, their combined application substantially improved diagnostic performance, achieving an overall sensitivity of 96.2%. This finding highlights the importance of integrating different endoscopic approaches to overcome the inherent limitations of each modality. However, these results should be interpreted cautiously, as they were derived from a relatively small subgroup of 26 patients and also formal paired statistical comparisons between EUS-FNB and cholangioscopy were not performed. Therefore, these findings should be considered exploratory and hypothesis-generating. Prospective multicenter studies are required to validate these observations.

The differences observed between EUS and cholangioscopy reflect the biological and anatomical heterogeneity of biliary malignancies. EUS demonstrated particular value in patients with discrete mass lesions, allowing tissue acquisition from primary tumors and suspicious lymph nodes while simultaneously providing locoregional staging. In contrast, digital cholangioscopy was particularly useful for infiltrative biliary strictures, where direct visualization of the ductal mucosa and targeted biopsy facilitated diagnosis in lesions lacking a well-defined extrabiliary mass. Notably, the sensitivity of visual assessment exceeded that of biopsy alone in our cohort, emphasizing that characteristic cholangioscopic features can provide important diagnostic information even when histological confirmation is challenging.

Our findings are consistent with previously published studies demonstrating good but incomplete diagnostic performance for both EUS-guided tissue acquisition and cholangioscopy when performed independently. However, evidence evaluating their combined use remains limited. The marked improvement in sensitivity observed in our cohort suggests that a multimodal endoscopic strategy may substantially reduce false-negative diagnoses, particularly in patients with complex hilar strictures or persistent diagnostic uncertainty after conventional ERCP-based sampling. Rather than applying a uniform diagnostic approach, our results support tailoring the choice of endoscopic modality according to lesion morphology, anatomical location, and the specific clinical question.

An important consideration is the safety of combining advanced endoscopic techniques. In our cohort, both procedures were safe, with no severe adverse events or deaths. Cholangioscopy showed a higher complication rate, mainly mild cholangitis, occasional pancreatitis, and bleeding, while EUS-related issues were limited to mild pancreatitis. These results are consistent with previous studies indicating a slightly higher risk of infectious complications after cholangioscopy, likely due to prolonged intraductal instrumentation and irrigation. All adverse events were managed conservatively without long-term effects, confirming the safety of both procedures in experienced centers with proper precautions.

Several limitations of our institutional experience should be acknowledged. This was a retrospective, single-center study performed at a tertiary referral institution with a predominance of complex perihilar lesions, which may limit the generalizability of the findings. In addition, the choice of diagnostic modality was individualized according to lesion characteristics and clinical judgment rather than a standardized protocol, introducing the potential for selection bias. Consequently, the reported diagnostic performance of EUS-guided tissue acquisition and digital cholangioscopy should not be interpreted as a direct head-to-head comparison. The number of patients undergoing both procedures was relatively small (n = 26); therefore, the high diagnostic sensitivity observed in this subgroup should be considered exploratory and hypothesis-generating. Furthermore, detailed procedural characteristics, including the type and gauge of the FNB needle, were not consistently available because of the retrospective design, and adverse events were not graded using a standardized classification system. Nevertheless, our cohort reflects real-world clinical practice and represents the patient population in whom diagnostic uncertainty is greatest and multimodal endoscopic evaluation is most likely to provide meaningful clinical benefit. Prospective multicenter studies using standardized diagnostic algorithms are warranted to validate these findings.

Overall, our experience complements the existing literature by demonstrating that the greatest diagnostic yield is achieved when EUS and digital cholangioscopy are integrated rather than used in isolation (**Table 2**). As precision oncology continues to evolve, combining high-resolution imaging, targeted tissue acquisition, accurate locoregional staging, and direct intraductal assessment is likely to become the preferred diagnostic strategy for patients with suspected biliary malignancies.

**Table 2:** Diagnostic performance of combined endoscopic tissue acquisition strategies for suspected malignant biliary strictures.

*Study*	Combined strategy	EUS sensitivity	Second modality sensitivity	Combined sensitivity
*Moura et al., 2020* (42)	EUS-FNA + ERCP-based tissue sampling	**76%**	**58%**	**86%**
*Lee et al., 2019* (56)	EUS-FNA + cholangioscopy-guided biopsy	**75%**	**77%**	**100%**
*Our study*	EUS-FNB + digital cholangioscopy	**65.0%**	**72.2%**	96.2%*

*Combined sensitivity was calculated in the subgroup of 26 patients who underwent both EUS-FNB and digital cholangioscopy and should be interpreted as exploratory owing to the limited sample size.

Bold values indicate the highest reported sensitivity within each study.

## 6 Future perspectives

The diagnostic landscape of biliary malignancies is evolving rapidly, driven by advances in imaging, molecular biology, and computational technologies. While EUS-guided tissue acquisition and digital cholangioscopy have substantially improved diagnostic accuracy, future progress will likely depend on integrating multiple complementary diagnostic platforms rather than further refining a single technique. The transition toward precision oncology is shifting the focus from establishing a histological diagnosis alone to obtaining comprehensive biological information that can guide individualized treatment strategies (43).

Artificial intelligence (AI) is expected to play an increasingly important role in endoscopic diagnosis. Machine learning algorithms are being developed to assist in interpreting EUS images, identify malignant cholangioscopic features, and reduce interobserver variability. Early studies suggest that AI-assisted image analysis may improve lesion detection, characterize indeterminate strictures with greater accuracy, and support real-time decision-making during endoscopic procedures (57).

Emerging imaging technologies may further complement current endoscopic techniques. Probe-based confocal laser endomicroscopy (pCLE) enables real-time microscopic assessment of the biliary epithelium during ERCP and has shown promise in improving the characterization of indeterminate strictures (58). Likewise, radiomics and radiogenomic approaches are increasingly being explored to extract quantitative imaging biomarkers from CT, MRI, and EUS examinations (59). By integrating imaging features with molecular and clinical data, these techniques may improve risk stratification and help predict tumor biology before tissue confirmation.

Liquid biopsy represents another promising area of research. Analysis of circulating tumor DNA (ctDNA), circulating tumor cells, microRNAs, and extracellular vesicles offers the potential for minimally invasive diagnosis, molecular profiling, and longitudinal monitoring of treatment response (60). Although these technologies are not yet incorporated into routine clinical practice, they may complement tissue-based diagnostics, particularly in patients with limited tissue availability or unresectable disease.

Ultimately, the future of biliary oncology lies in the development of integrated diagnostic algorithms that combine high-resolution imaging, endoscopic visualization, targeted tissue acquisition, molecular profiling, and computational decision-support tools (61, 62). Rather than relying on a single diagnostic modality, personalized diagnostic pathways tailored to lesion morphology, anatomical location, and molecular characteristics are likely to become the standard of care.

## 7 Conclusions

The diagnosis of biliary malignancies remains one of the greatest challenges in gastrointestinal oncology because of the heterogeneous biological behavior of cholangiocarcinoma, its frequently infiltrative growth pattern, and the limited diagnostic yield of conventional tissue acquisition techniques. Although significant advances have been made in endoscopic imaging and biopsy methods, no single modality can comprehensively address all aspects of disease characterization.

Endoscopic ultrasound and digital cholangioscopy have transformed the diagnostic approach to biliary strictures by providing complementary information. EUS offers high-resolution evaluation of the extrabiliary component of the disease, enabling accurate locoregional staging and tissue acquisition from primary tumors, lymph nodes, and metastatic lesions. In contrast, digital cholangioscopy provides direct intraductal visualization and targeted biopsy, improving the evaluation of superficial and infiltrative biliary lesions that are often difficult to diagnose using conventional techniques. Rather than representing competing approaches, these modalities should be viewed as complementary tools within a unified diagnostic pathway.

Our institutional experience suggests that integrating EUS-guided tissue acquisition and digital cholangioscopy may improve diagnostic confidence in selected patients with suspected biliary malignancies. Given the limited size of the combined-modality subgroup, these findings should be considered preliminary and require validation in larger prospective studies.

They do, however, reinforce the importance of tailoring the diagnostic strategy to the anatomical location, morphology, and clinical presentation of each lesion, rather than relying on a single endoscopic technique.

Looking ahead, the integration of molecular diagnostics, next-generation sequencing, artificial intelligence, digital pathology, radiomics, and liquid biopsy is expected to further refine the diagnostic pathway for biliary malignancies. Future strategies will increasingly combine advanced imaging, targeted tissue acquisition, and comprehensive molecular profiling to enable earlier diagnosis, more accurate staging, and personalized therapeutic decision-making.

Ultimately, the future of biliary oncology lies in a multimodal, precision-based approach that integrates complementary endoscopic techniques with emerging molecular and computerized technologies. Such an approach has the potential to improve diagnostic confidence, optimize patient selection for therapy, and ultimately enhance clinical outcomes in this complex and challenging disease.

## Data Availability

The datasets analyzed in this study are not publicly available because they contain potentially identifiable clinical information. De-identified data may be made available by the corresponding author upon reasonable request and subject to institutional and ethical approval.
